# ExCyto PCR Amplification

**DOI:** 10.1371/journal.pone.0012629

**Published:** 2010-09-08

**Authors:** Vinay Dhodda, Ronald Godiska, Jeffrey D. VanWye, David Mead, Rebecca Hochstein, Lynne Sheets, Sarah Vande Zande, Chris Niebauer, Douglas L. Crawford, Marjorie F. Oleksiak

**Affiliations:** 1 Lucigen Corporation, Middleton, Wisconsin, United States of America; 2 Rosenstiel School of Marine and Atmospheric Science, Division of Marine Biology and Fisheries, University of Miami, Miami, Florida, United States of America; University of Wisconsin-Milwaukee, United States of America

## Abstract

**Background:**

ExCyto PCR cells provide a novel and cost effective means to amplify DNA transformed into competent bacterial cells. ExCyto PCR uses host *E. coli* with a chromosomally integrated gene encoding a thermostable DNA polymerase to accomplish robust, hot-start PCR amplification of cloned sequences without addition of exogenous enzyme.

**Results:**

Because the thermostable DNA polymerase is stably integrated into the bacterial chromosome, ExCyto cells can be transformed with a single plasmid or complex library, and then the expressed thermostable DNA polymerase can be used for PCR amplification. We demonstrate that ExCyto cells can be used to amplify DNA from different templates, plasmids with different copy numbers, and master mixes left on ice for up to two hours. Further, PCR amplification with ExCyto cells is comparable to amplification using commercial DNA polymerases. The ability to transform a bacterial strain and use the endogenously expressed protein for PCR has not previously been demonstrated.

**Conclusions:**

ExCyto PCR reduces pipetting and greatly increases throughput for screening EST, genomic, BAC, cDNA, or SNP libraries. This technique is also more economical than traditional PCR and thus broadly useful to scientists who utilize analysis of cloned DNAs in their research.

## Introduction

DNA amplification plays a critical role in many molecular biology procedures [1]. Molecular analysis of thousands of genes and DNA templates is now routine, due to the advent of the polymerase chain reaction (PCR) [2], [3], [4] and the development of high-throughput sequencing technology. For genome sequencing projects, the recombinant DNA template typically is purified from the host cell and then amplified by conventional PCR using a highly purified thermostable DNA polymerase. Alternately, colony PCR can be performed by adding a DNA polymerase with a PCR master mix to a lysed cell sample from a single recombinant colony, omitting the step of template purification. For high-throughput approaches, this method requires adding the purified DNA polymerase to a master mix and preventing non-specific amplification by hot-start or other approaches.

In this manuscript we describe novel methods, which utilize *E. coli* cells (“ExCyto PCR” cells) that express a chromosomally integrated gene for a thermostable DNA polymerase (tsDNA polymerase), facilitating robust PCR amplification without the addition of exogenous enzyme. Because the tsDNA polymerase is integrated into the chromosome of the ExCyto PCR cells, one can transform these cells with plasmids or BACs and maintain the expression of the tsDNA polymerase. The stable integration of the tsDNA polymerase is functionally different from co-expressing the tsDNA polymerase using a plasmid because it is difficult maintain multiple, different plasmids in *E. coli* cells. When ExCyto PCR cells are transformed with cloned DNAs in a single plasmid or complex library, the cloned DNA can be PCR amplified with the simple addition of buffer, nucleotides, and template specific primers, but without the addition of enzyme. This approach significantly reduces the cost of DNA amplification, removes major bottlenecks in template purification, and simplifies automation, thereby reducing technical errors.

## Results and Discussion

**Figure 1:**
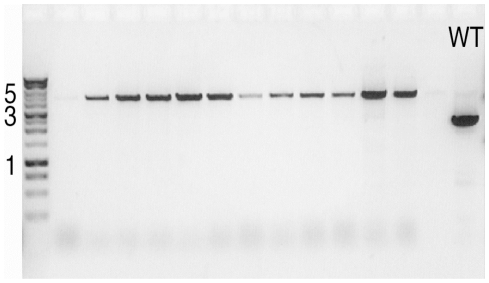
ExCyto PCR of chromosomal integrants. Two µl of overnight cultures from chromosomal integrants were used for PCR with primers flanking the melAmelB bicistron integration site. Integrants show a band at 4.5 kb. Wild type (WT, last lane) has a band at 2 kb. One kb molecular weight markers are shown in the first lane.

We stably integrated a thermostable DNA polymerase into *E. coli* cells and tested whether the resulting cells could be used for robust PCR of plasmid DNAs propogated in the same cells without the addition of exogenous DNA polymerase. Chromosomal integration of the DNA polymerase into ExCyto cells was confirmed by performing ExCyto PCR analysis of the integration site (Figure 1): amplification reactions contained primers flanking the integration site, dNTPs, and buffer, but no exogenous polymerase. Chromosomal integrants yielded a 4.5 kb band, in contrast to the 2 kb wild type gene (Figure 1).

**Figure 2 pone-0012629-g002:**
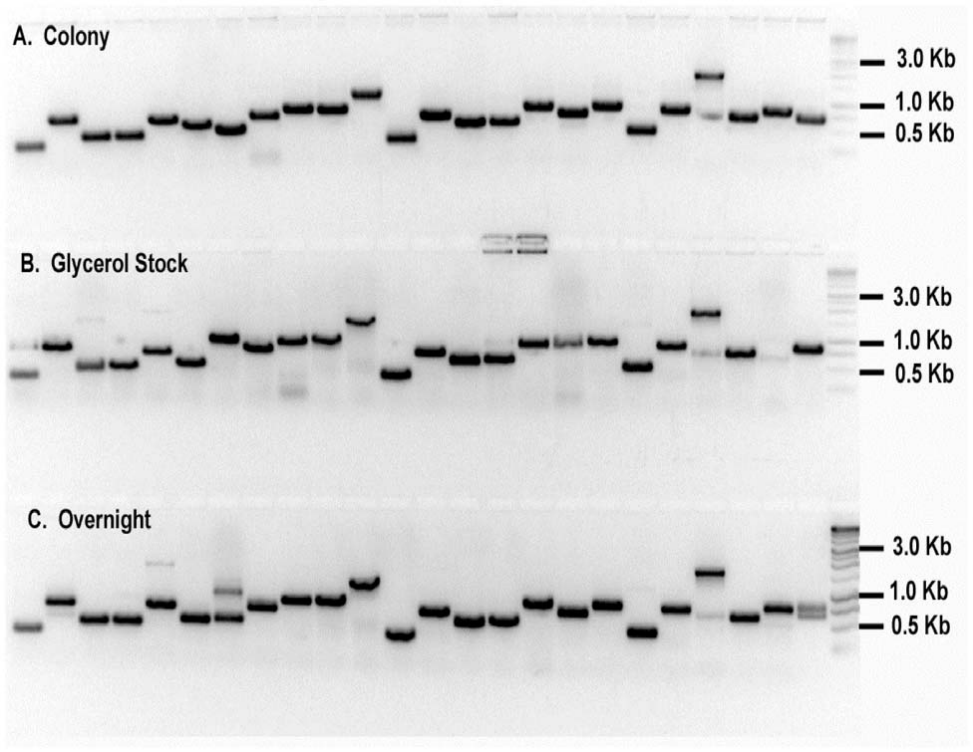
ExCyto PCR amplification. A cDNA library was transformed into ExCyto cells. Twenty-four different cDNAs were amplified without addition of exogenous polymerase using (A) single colonies, (B) 2 µl of frozen glycerol stocks, or (C) 2 µl of overnight cultures. One kb molecular weight markers are shown in the last lane.

To assay the effectiveness of ExCyto PCR, shotgun genomic DNA and cDNA libraries were transformed into both chemically competent and electrocompetent ExCyto cells, and then the cloned DNAs were amplified from single colonies (Figure 2A), 2 µl of frozen glycerol stocks (15% glycerol stored at −80°C, Figure 2B), or 2 µl of overnight cultures (Figure 2C). ExCyto PCR amplified inserts from approximately 300 to 3,000 bp from single colonies, glycerol stocks, and overnight cultures equally well based on the resulting PCR products (Figure 2). Thus, ExCyto PCR cells are useful for both actively growing and stored bacterial cultures.

**Figure 3 pone-0012629-g003:**
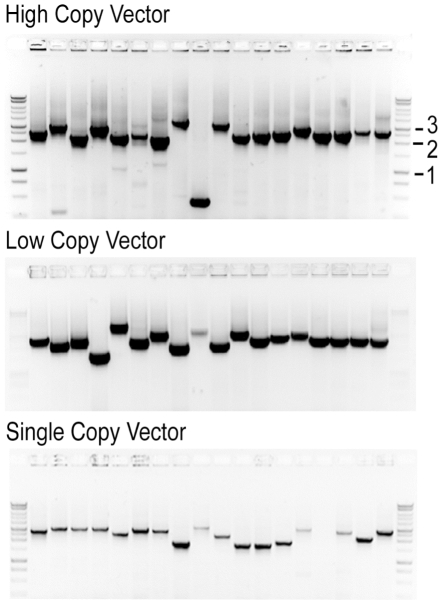
ExCyto PCR and plasmid copy numbers. Clones from a shotgun library of genomic DNA transformed into ExCyto cells were grown overnight, and 2 ul of the overnight cultures were amplified with ExCyto PCR from the (A) pEZSEQ high copy vector (∼300 copies/cell), (B) pSMART GC low copy vector (approx. 20 copies/cell), and (C) pSMART VC single-copy vector (1 copy per cell). In total, 54 different genomic DNA clones were amplified. One kb molecular weight markers are shown in the first and last lanes.

To test the effect of template copy number on ExCyto PCR amplification, we amplified clones from a random genomic DNA library in high, low, and single copy vectors (Figure 3). Amplification worked well even with a single copy of plasmid per cell. However, amplification was more robust using high and low copy vectors as template. Thus, use of high and low copy vectors rather than single copy vectors should depend on the purpose of the PCR amplification (*e.g.*, simple diagnostics of presence/absence *versus* amplification of a probe).

**Figure 4 pone-0012629-g004:**
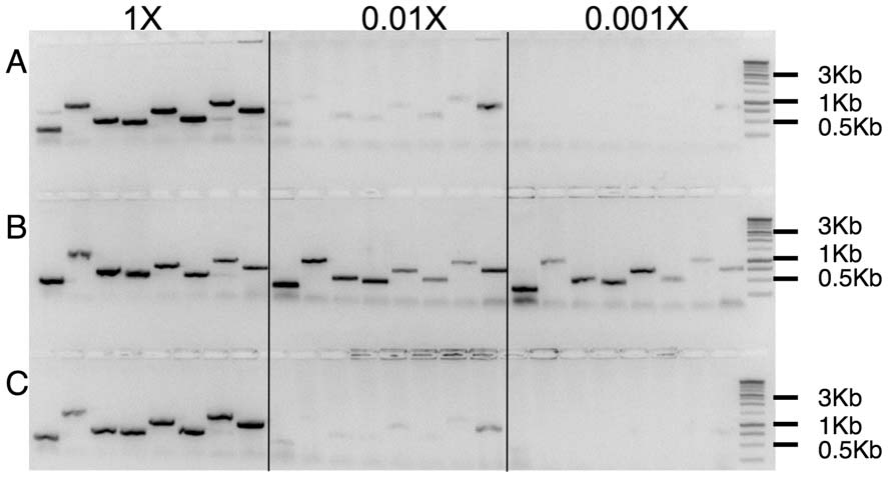
Dilution series of ExCyto PCR cells. Eight different clones from a cDNA library transformed into ExCyto cells were grown overnight, and a serial dilution was used for amplification (undiluted, 100-, and 1000-fold dilutions). In (A), bacteria were diluted, reducing both the target DNA and the tsDNA polymerase. In (B), to compensate for the serial dilution of the tsDNA polymerase, 2 ul of ExCyto cells (OD 1.1) without plasmids was added. In (C), to compensate for the serial dilution of plasmid, 2 ul of bacteria with the same plasmid, but without the tsDNA polymerase was added. A 10-fold dilution is not shown, but gives similar amplification to that seen with undiluted cells. One kb molecular weight markers are shown in the last lane.

To further test the sensitivity of ExCyto cells, ExCyto PCR was done on serial dilutions (1, 1/10, 1/100, and 1/1,000) of overnight cultures (OD ≈1.0–1.2). Three sets of amplifications were done (Figure 4): the first was a serial dilution of bacteria, which reduces both the concentration of target DNA and of tsDNA polymerase (Figure 4A), the second was a serial dilution with a constant amount of tsDNA polymerase maintained by adding 2 ul of ExCyto PCR cells without plasmid (Figure 4B), and the third was a serial dilution of bacteria keeping the target amount constant (by adding 2 ul of bacteria with plasmid but not tsDNA polymerase, Figure 4C). Although the 100-fold dilution had an amplified product, there was a significant decrease because of the reduced amount of tsDNA polymerase (compare 4B to 4A or 4C). This suggests that the amplification is dependent on the expression of tsDNA polymerase in ExCyto cells.

**Figure 5 pone-0012629-g005:**
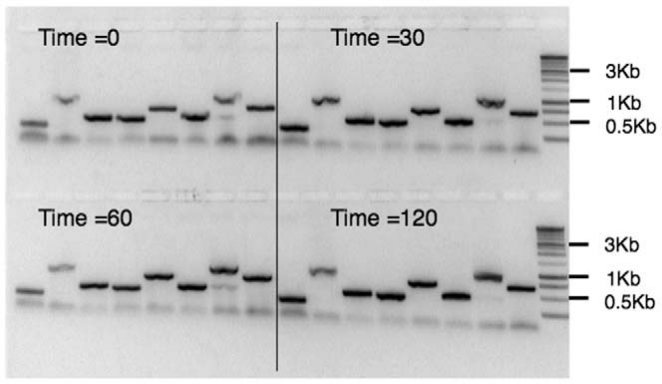
Robustness of ExCyto PCR. Eight different clones from a cDNA library transformed into ExCyto cells were grown overnight, and 2 ul of the overnight cultures were added to reaction mix and left on ice for 0, 30, 60 and 120 minutes prior to ExCyto PCR amplification. One kb molecular weight markers are shown in the last lane.

For high throughput approaches, it may be necessary to accumulate many plates to process them simultaneously. To test how robust ExCyto PCR was if the reaction mix had to wait before use, we added 2 ul of ExCyto PCR cells to the reaction mixes and let them sit on ice for 30, 60, and 120 minutes (Figure 5). There was no difference in the amount of product with increasing incubation time on ice. The specificity and level of production is similar among time points, supporting the concept that ExCyto PCR is similar to hot-start PCR. That is, because the bacterial cell membrane separates primers and dNTPs from polymerase and target DNA, PCR is not initiated until the reaction is heated, effectively creating a hot start.

**Figure 6:**
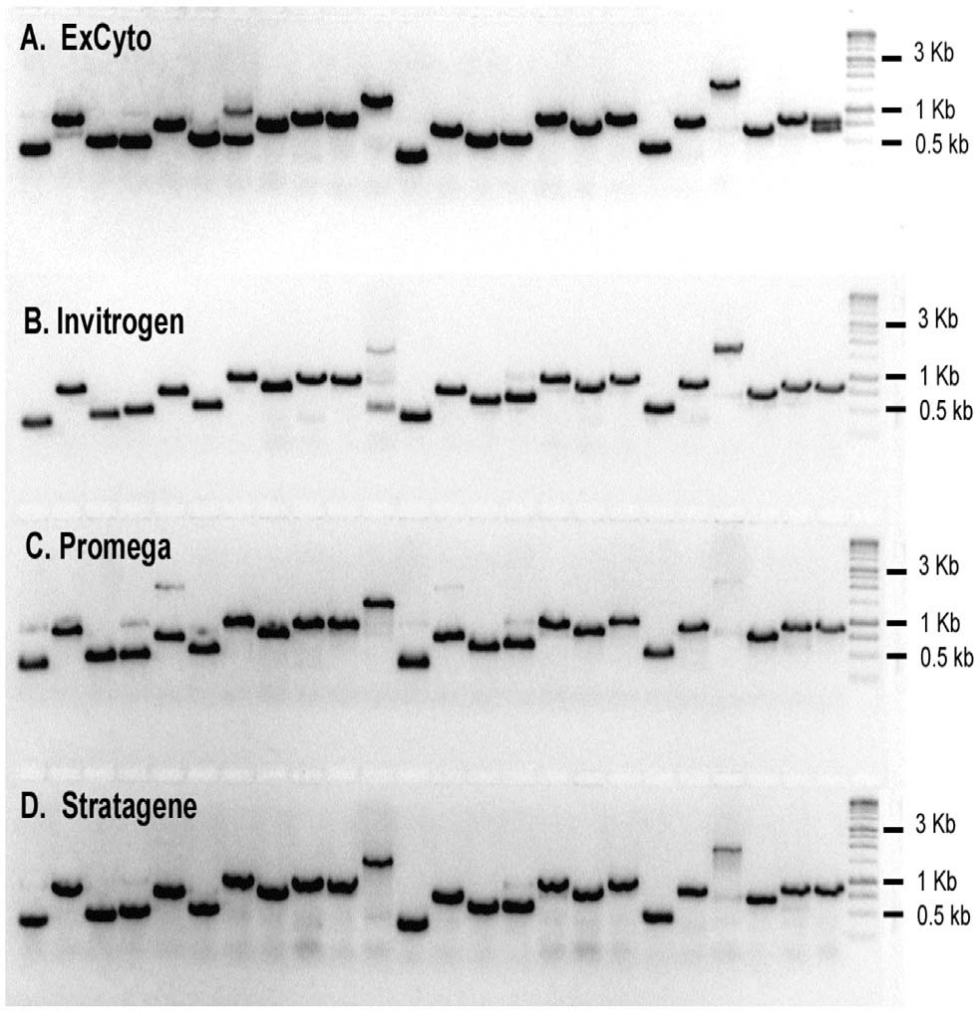
ExCyto *versus* standard PCR. (A) Twenty-four different clones from a cDNA library transformed in ExCyto cells were grown overnight, and 2 µl of overnight cultures were used for ExCyto PCR amplification. (B, C, & D) Standard PCR on 2 µl of overnight cultures from the same 24 clones as in (A) above, except clones were transformed into parent cells without the integrated thermostable polymerase. The cDNA inserts were amplified using Invitrogen's Taq DNA Polymerase (B), Promega's GoTaq DNA Polymerase (C), or Stratagene's Taq DNA Polymerase (D). One kb molecular weight markers are shown in the last lane.

To compare ExCyto PCR to conventional PCR, the same plasmids were transformed into ExCyto cells or the parental *E. coli* cells. The cDNA inserts were amplified from 2 µl of an overnight culture as template, using ExCyto PCR or conventional PCR with addition of commercially available, purified DNA polymerases (1.25 to 2.5 units) and their recommended buffers. Primers and amplification conditions were identical for the ExCyto PCR and conventional PCR. Amplification products from ExCyto PCR were comparable to or better than amplifications produced by exogenously added DNA polymerases (Invitrogen, Promega, or Stratagene; Figure 6). The yields of purified amplification products were quantified spectrophotometrically and were not statistically different for ExCyto PCR or PCR using Invitrogen, Promega, and Stratagene polymerases (p>0.10, ANOVA). Thus, ExCyto PCR provided a simpler but equally effective means to amplify cloned DNAs of a range of sizes up to 3 kb.

Amplified DNA is the backbone of many genomic analyses. It is used for sequence analyses, protein expression, and microarray analyses. Most of the cost of amplifying DNA is due to the cost of the PCR enzyme and the pipette tips used to aliquot reagents. As shown here, ExCyto PCR can significantly reduce the cost by removing the need to purchase and aliquot the thermostable polymerase. Importantly, amplification using ExCyto PCR is robust (Figures 2 & 6) and yields PCR fragments up to 3,000 bp from single colonies, bacterial cultures, glycerol stocks, and low copy plasmids. ExCyto PCR is sensitive enough to amplify a single copy plasmid (Figure 3) or a dilution corresponding to approximately 0.1 plasmid per cell (Figure 4).

In addition to cost savings, ExCyto PCR provides significant improvements for automation. There is no need to add enzyme to a PCR master mix; thus, plates can be filled in bulk with master mix and stored frozen or lyophilized prior to amplification. The presence of the tsDNA polymerase gene on the chromosome rather than on a plasmid eliminates a possible source of contamination. Furthermore, because the polymerase is isolated in the bacterial cells, this technique provides a built-in “hot start” for PCR. Thus, plates filled with master mix in bulk can be inoculated with ExCyto PCR cells in bulk prior to initiating amplification. Importantly, ExCyto PCR cells can be added to a master mix for at least 2 hours before initiating thermal cycles (Figure 5). Thus, many plates of amplification can be set up prior to processing.

ExCyto cells will allow many laboratories to amplify DNAs from genomic shotgun libraries or cDNA libraries by simply adding a single colony of bacteria or 2 µl of overnight culture to a stock master mix of dNTPs, buffer, and vector specific primers, without the addition of exogenous enzymes. Thus, PCR master mixes can be stocked in a freezer, and the reaction can be initiated by only adding a bacterial culture or colony picks, thereby simplifying automation, reducing technical errors and further decreasing costs. This system greatly reduces costs and effort, yielding amplified DNA that is readily used for sequencing, microarray printing, or other molecular processes.

## Materials and Methods

ExCyto cells were created by integrating a construct consisting of the gene for Taq DNA polymerase, under control of the tac promoter, along with a gentamycin resistance gene. In addition, each end of the construct contained 500 bp of sequence homologous to the *mel*A-*mel*B region of the *E. coli* chromosome, allowing integration using the bacteriophage lambda RED recombinase system [5]. The *mel*A-*mel*B locus was used as an integration target because it provides a visual screen for integration: when grown on 5-Bromo-4-chloro-3-indolyl-alpha-D-galactopyranoside medium, wild type cells are blue, but *mel*A-*mel*B knockouts are white. The *E. coli* strain used for stable integration was DH10B. Transformants were selected on gentamycin plates, and the recombinase plasmid was eliminated from the host by growing at 42°C.

Chemically competent cells were prepared by growth to log phase in LB and resuspension in TB (10 mM Pipes, 55 mM MnCI_2_, 15 mM CaC1_2_, 250 mM KCI), as described [6]. Plasmid DNAs were mixed with thawed cells, and cells were heat shocked at 42°C for 30 seconds, allowed to recover in 1 ml of SOC (2% Bacto tryptone, 0.5% Bacto yeast extract, 10 mM NaCl, 2.5 mM KCl, 10 mM MgCl_2_ 10 mM MgSO_4_, and 20 mM glucose) with shaking at 225 RPM for 1 hour at 37°C, and plated on selective plates. Electrocompetent cells were prepared by growth to log phase in LB and washing with H_2_O as described [7]. Cells were thawed on ice, mixed with 1 ul of plasmid DNA (∼50 ng) and electroporated at 1.7–1.9 kV using a BTX ECM 399 electroporation system. Cells were allowed to recover in 1 ml of SOC with shaking at 225 RPM, for 1 hour at 37°C before plating on selective plates.

For all PCR reactions, we used biological replicates rather than technical replicates in order to assess the robustness of the reactions using a variety of different templates. Thus, templates ranged in size from ∼300 bp to ∼4500 bp and were amplified from different plasmids or genomic DNA (Figures 1–6). Plasmids were single copy, low copy, and high copy (Figure 3). In total, we used ExCyto PCR to amplify 111 different templates. ExCyto PCR activity was assessed by gel electrophoresis on 1% TBE-agarose gels and was evaluated based on the intensity of the bands across biological replicates. We did not directly quantify DNA polymerase expression.

To compare amplification using ExCyto PCR cells to amplification using commercial DNA polymerases, the same 24 plasmids were amplified using ExCyto PCR and three commercial, DNA polymerases (Figure 6). PCR products were purified using magnetic beads [8] in order to get rid of unincorporated nucleotides and primers and quantified with a spectrophotometer. Yields among ExCyto cells and commercial DNA polymerases were analyzed using analysis of variance (ANOVA).

DNA in ExCyto PCR cells was amplified from single colonies, 2 µl of frozen glycerol stocks, 2 µl of overnight cultures or a serial dilution of overnight cultures (OD ≈1.0–1.2, ∼1.6–1.9×10^6^ cells). Single colonies were picked using plastic pipette tips, transferred to 50 µl of PCR master mix lacking enzyme (50 mM Tris HCl, pH 9.2, 16 mM NH_4_2SO_4_, 2.25 mM MgCl_2_, 0.1% Tween 20, 2% DMSO, 0.2 uM primers, and 0.2 mM dNTPs), and PCR amplified using a MJ Research, Model PTC-200. The remaining bacteria on the tips were transferred to 2X YT bacterial culture media. After overnight growth, 2 µl was transferred to 50 µl of PCR master mix lacking enzyme and PCR amplified. The remaining overnight culture was used to make a 15% glycerol stock, and 2 µl of these stocks were similarly amplified. PCR was performed using 30 cycles of 94°C for 15 seconds, 62°C (Tm-10°C) for 15 seconds, and 72°C for 1 minute per kb, followed by a final extension at 72°C for 10 minutes. Reactions were held at 4°C prior to gel analysis. Unless stated, the vector used in the experiments was pSMART® cDNA-SS4 (Lucigen), and the primer sequences were:

SF22: 5′ TTA ATA CGA CTC ACT ATA AGG GGT GTG G 3′ and

SR22: 5′ GCC TCC GGT CGG AGG CTT TTG ACT TTC TGC TAT GGA GG 3′.

For the pSMART VC single-copy vector, the primer sequences were:

BSM-F: 5′AAA GAA GGA AAG CGG CCG CCA GG 3′

BFR-R: 5′CTA TAC GAA GTT ATG TCA AGC GG 3′

For the pSMART GC low copy vector, the primers sequences were:

SL1: 5′ CAG TCC AGT TAC GCT GGA GTC 3′ and

SR2: 5′ GGT CAG GTA TGA TTT AAA TGG TCA GT 3′.

For the pEZSEQ high copy vector, the primer sequences were:

Z-Rev primer: 5′ AGC GGA TAA CAA TTT CAC ACA GGA 3′

Z-For primer: 5′ CGC CAG GGT TTT CCC AGT CAC GA 3′.
